# Subditine, a New Monoterpenoid Indole Alkaloid from Bark of *Nauclea subdita* (Korth.) Steud. Induces Apoptosis in Human Prostate Cancer Cells

**DOI:** 10.1371/journal.pone.0087286

**Published:** 2014-02-14

**Authors:** Sook Yee Liew, Chung Yeng Looi, Mohammadjavad Paydar, Foo Kit Cheah, Kok Hoong Leong, Won Fen Wong, Mohd Rais Mustafa, Marc Litaudon, Khalijah Awang

**Affiliations:** 1 Department of Chemistry, Faculty of Science, University of Malaya, Kuala Lumpur, Malaysia; 2 Department of Pharmacology, Faculty of Medicine, University of Malaya, Kuala Lumpur, Malaysia; 3 Department of Pharmacy, Faculty of Medicine, University of Malaya, Kuala Lumpur, Malaysia; 4 Department of Medical Microbiology, Faculty of Medicine, University of Malaya, Kuala Lumpur, Malaysia; 5 Institut de Chimie des Substances Naturelles, Centre National de la Recherche Scientifique, Gif-sur-Yvette, Cedex, France; National Health Research Institutes, Taiwan

## Abstract

In this study, a new apoptotic monoterpenoid indole alkaloid, subditine (**1**), and four known compounds were isolated from the bark of *Nauclea subdita*. Complete ^1^H- and ^13^C- NMR data of the new compound were reported. The structures of isolated compounds were elucidated with various spectroscopic methods such as 1D- and 2D- NMR, IR, UV and LCMS. All five compounds were screened for cytotoxic activities on LNCaP and PC-3 human prostate cancer cell-lines. Among the five compounds, the new alkaloid, subditine (**1**), demonstrated the most potent cell growth inhibition activity and selective against LNCaP with an IC_50_ of 12.24±0.19 µM and PC-3 with an IC_50_ of 13.97±0.32 µM, compared to RWPE human normal epithelial cell line (IC_50_ = 30.48±0.08 µM). Subditine (**1**) treatment induced apoptosis in LNCaP and PC-3 as evidenced by increased cell permeability, disruption of cytoskeletal structures and increased nuclear fragmentation. In addition, subditine (**1**) enhanced intracellular reactive oxygen species (ROS) production, as reflected by increased expression of glutathione reductase (GR) to scavenge damaging free radicals in both prostate cancer cell-lines. Excessive ROS could lead to disruption of mitochondrial membrane potential (MMP), release of cytochrome c and subsequent caspase 9, 3/7 activation. Further Western blot analyses showed subditine (**1**) induced down-regulation of Bcl-2 and Bcl-xl expression, whereas p53 was up-regulated in LNCaP (p53-wild-type), but not in PC-3 (p53-null). Overall, our data demonstrated that the new compound subditine (**1**) exerts anti-proliferative effect on LNCaP and PC-3 human prostate cancer cells through induction of apoptosis.

## Introduction

The Rubiaceae family (Madder family) is one of the largest of the angiosperms with more than 637 genera and almost 10,700 species [1]. The genus *Nauclea* which belongs to this family, consists of about 35 species worldwide [2] and in Malaysia, there are two *Nauclea* species; *N. officinalis* and *N. subdita* [3]. *Nauclea subdita* (Korth.) Steud. is a tropical plant that grows in lowland to hill forests, in swampy places and frequently along streams and rivers [4]. It is a small or medium tree to 25 m tall and 60 cm girth [3]. The plants from this genus are known to produce interesting monoterpenoid indole alkaloids with high structural diversity such as naucline [5], nauclealines B [6] and naufoline [7]. Many of them exhibited significant biological activities; anti-convulsant [8], anti-proliferative [9] and vasorelaxant activities [5].

Prostate cancer is the most frequently diagnosed cancer among men in the developed world. An estimated 238,590 new cases will be diagnosed and 29,720 deaths will result from prostate cancer in the United States in 2013 (Cancer Facts and Figures 2013, American Cancer Society, 2013). Although the mechanisms that drive prostate cancer have not been completely understood, age, race, and family history of the prostate cancer patients have been shown to be the potential factors closely associated with this fatal disease [10].

**Figure 1:**
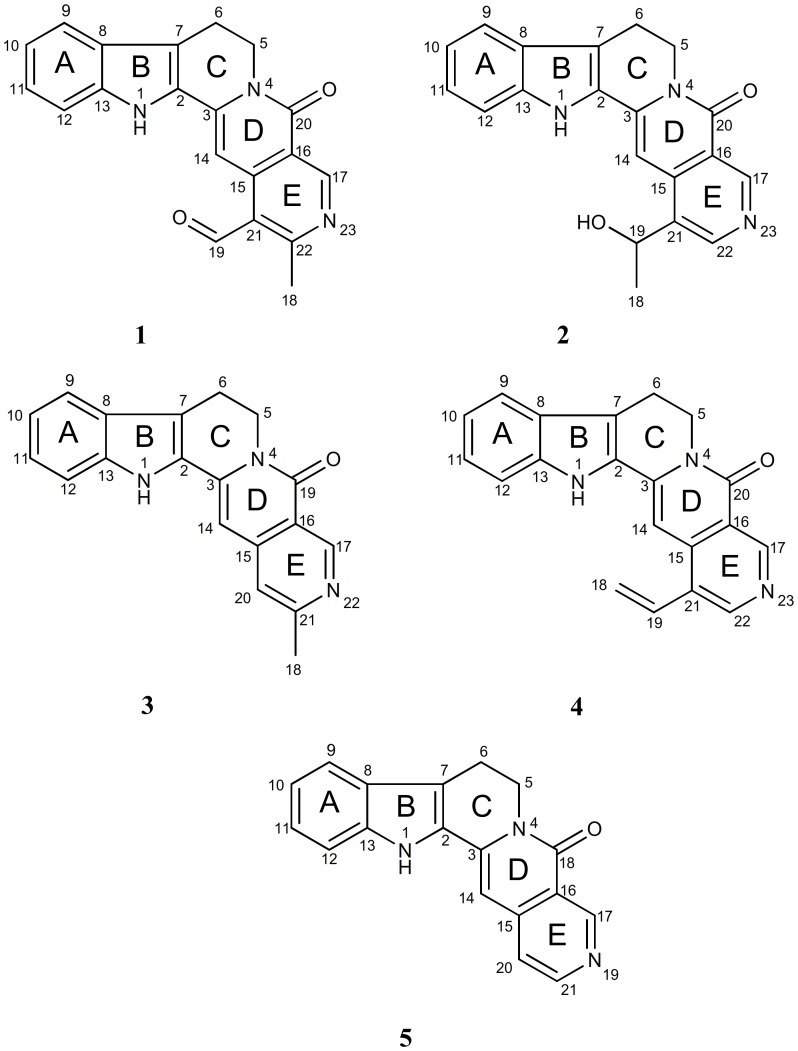
Chemical structure of subditine (1) angustoline (2), angustidine (3), angustine (4), nauclefine (5) isolated from the bark of *Nauclea subdita*. The structure of new compound, subditine (**1**) were elucidated using various spectroscopic method which were 1D-NMR (^1^H, ^13^C, DEPT), 2D-NMR (HSQC, HMBC, NOESY), UV, IR and LCMS while the structure of the other four known compounds were confirmed through the comparison of NMR data with literature values.

In our continuous effort to search for new and bioactive chemical constituents from the Malaysia flora [11]–[15], a new cytotoxic and apoptotic monoterpenoid indole alkaloid, subditine (**1**), has been isolated from the bark of *Nauclea subdita* together with the four known alkaloids; angustoline (**2**) [11], [16], [17], angustidine (**3**) [18], [19], angustine (**4**) [20], [21], nauclefine (**5**) [22], [23] (Figure 1). In the present paper, we report the isolation and characterization of subditine (**1**), the cytotoxic activities of alkaloids **1**–**5** as well as the apoptotic mechanism of **1** against human prostate cancer cells LNCaP and PC-3.

## Materials and Methods

### General Procedures

The 1D- and 2D-NMR were recorded in deuterated chloroform (CDCl_3_) (Merck, deuteration degree min. 99.8%) using JEOL LA 400 MHz FT NMR and JEOL ECA 400 MHz FT NMR spectrometer. The mass spectra were obtained on a Shimadzu LCMS-IT-TOF. The ultraviolet absorption spectra were obtained using Shimadzu UV-250 Ultraviolet-Visible Spectrometer. Solvent used was methanol (CH_3_OH). IR spectra were obtained on a Perkin Elmer Spectrum 400-FTIR Spectrometer with CHCl_3_ as solvent. All solvents, except those used for bulk extraction are AR grade. Silica gel 60 (Merck, 0.040–0.063 mm) was used for column chromatography (CC). Aluminium supported silica gel 60 F_254_ plates 20×20 cm were used for thin layer chromatography (TLC) (Merck, Germany). Preparative thin layer chromatography (PTLC) silica gel 60 F_254_ glass plates 20×20 cm (Merck, Germany) were used for separation of compounds that cannot be separated by conventional column. TLC spots were visualized under UV light (254 and 365 nm) followed by spraying with Dragendorff’s reagent for alkaloid detection. A positive test result was indicated by the formation of orange spots.

### Plant Material

The bark of *Nauclea subdita* was collected at Hutan Simpan Bukit Kinta, Chemor, Perak, Malaysia by the phytochemical group of the Department of Chemistry, Faculty of Science, University of Malaya. The voucher specimens (KL 5254) of these plants were deposited at the Herbarium of the Department of Chemistry, University of Malaya, Kuala Lumpur, Malaysia. Plant collection have been approved by the head of Jabatan Perhutanan Negeri Perak (Perak State Forestry Department). The field studies did not involve endangered or protected species.

### Extraction and Isolation

Dried, grounded bark of the plant (1.7 kg) was first defatted with hexane (17 litres) for 3 days at room temperature. The hexane extract was filtered and dried at room temperature. Then the dried plant materials were moistened with ammonia solution and soaked for 2 hours. They were re-extracted with CH_2_Cl_2_ (17 litres) twice for a 3 day period. The supernatant obtained was concentrated using rotary evaporator under reduced pressure to a volume of 500 mL and examined for its alkaloid content (using TLC and confirmed by spraying with Dragendorff’s reagent). The extract was finally concentrated to give dichloromethane crude extract (5.0 g). The crude extract was subjected to CC over silica gel 60 using CH_2_Cl_2_ and MeOH solvent (100∶0, 99∶1, 98∶2, 97∶3, 96∶4, 95∶5, 94∶6, 90∶10, 83∶17, and 75∶25) and finally with 100% MeOH was used as eluent. By comparing TLC patterns of these fractions, fifteen fractions were finally obtained.

### Purification of Compound

Further purification of fraction 5 by PTLC yielded alkaloid **1** (10.6 mg, MeOH-CH_2_Cl_2_; 98∶2: saturated with NH_4_OH). Both known compounds of **3** (5.5 mg, MeOH-CH_2_Cl_2_; 98∶2: saturated with NH_4_OH) and **5** (6.2 mg, MeOH-CH_2_Cl_2_; 98∶2: saturated with NH_4_OH) were obtained after purification by PTLC from fraction seven while compounds **2** (7.5 mg, MeOH-CH_2_Cl_2_; 95∶5: saturated with NH_4_OH) and **4** (12.5 mg, MeOH-CH_2_Cl_2_; 98∶2: saturated with NH_4_OH) were obtained from fraction of twelve and six respectively.

### Alkaloid 1

Yellowish amorphous solid; UV (MeOH) λ_max_ (log ε): 393, 377, 210 nm; IR (CHCl_3_) ν_max_: 3430, 1640 cm^−1^; for ^1^H- and ^13^C-NMR spectroscopic data, see Table 1; LCMS -IT-TOF at *m/z* 330.1018 [M+H]^+^ for C_20_H_15_N_3_O_2_ (Calcd. for C_20_H_15_N_3_O_2_∶330.1237).

**Table 1:** ^1^H-NMR (400 MHz) and ^13^C-NMR (100 MHz) Spectral Data of Subditine (**1**) and Angustidine* (**3**) in CDCl_3_ and DMSO-*d_6_* respectively.

Position	^1^H		^13^C		HMBC of Subditine
	δ_H_ (multiplicity, *J* in Hz)		δ_C_		
	Subditine	Angustidine*	Subditine	Angustidine*	
NH-1	8.94 (br s)	11.82 (s)	–	–	–
2	–	–	127.3	127.7	–
3	–	–	139.4	137.0	–
5	4.51 (t, 6.9)	4.38 (t, 6.8)	40.5	40.3	3, 6, 7, 20
6	3.16 (t, 6.9)	3.11 (t, 6.5)	19.7	19.3	2, 5, 7
7	–	–	117.1	114.6	–
8	–	–	125.8	125.5	–
9	7.62 (d, 7.8)	7.62 (d, 8.0)	119.9	119.7	11, 13
10	7.19 (dd, 7.8, 7.1)	7.09 (t, 7.6)	120.9	119.9	11, 12
11	7.3 (dd, 8.2, 7.1)	7.23 (t, 7.2)	125.7	124.4	9, 13
12	7.47 (d, 8.2)	7.45 (d, 8.4)	111.9	112.0	10, 11
13	–	–	138.7	138.5	–
14	7.97 (s)	6.94 (s)	94.7	97.0	3, 16, 21
15	–	–	141.1	141.9	–
16	–	–	119.3	119.9	–
17	9.57 (s)	9.21 (s)	155.2	150.2	15, 16
18	2.98 (s)	2.58 (s)	22.6	24.3	22
19	10.72 (s)	–	192.6	160.2	21
20	–	7.35 (s)	161.7		–
21	–	–	127.6	117.2	–
22	–	–	165.9	145.0	–

^*^Literature values from Abreu and Pereira (1998).

### Cell Culture

Human prostate normal cell line (RWPE-1) and human prostate cancer cell lines; LNCaP and PC-3, were purchased from the American Type Culture Collection (ATCC, Manassas, Virginia, USA). LNCaP and PC-3 cells were grown in Roswell Park Memorial Institute medium (RPMI) supplemented with 10% heat-inactivated fetal bovine serum (FBS, Sigma-Aldrich, St. Louis, MO), 1% penicillin and streptomycin. RWPE-1 cells were maintained in Keratinocyte Serum Free Medium (K-SFM, ATCC) supplemented with bovine pituitary extract (BPE) and human recombinant epidermal growth factor (EGF). Mediums were supplemented with 10% heat-inactivated fetal calf serum (Sigma.), 100 U/ml penicillin and 100 mg/ml streptomycin (Flowlab, Sydney, Australia). All cells were maintained in a humidified atmosphere of 5% CO_2_ in air at 37°C incubator.

### Cell Proliferation Assay

The anti-proliferative activity was evaluated by performing MTT assays as previously described with minor modifications [24]. Briefly, cells were seeded 24 hours prior to treatment in a 96-well plate at 5×10^4^ cells/well in order to obtain 70% to 80% confluent cultures. The compounds were dissolved in DMSO (Sigma Chemical Co., St. Louis, Missouri, USA) followed by a 2×serial dilution for 10 points ranged from 0.825 µM to 100 µM. The 96-well plate was incubated for 24 hours at 37°C in a humidified atmosphere with 5% CO_2_. At the end of incubation, 50 µl of MTT solution (2 mg/ml; Sigma) was added to each well. The plate was then incubated for 4 hours. All medium was removed and the purple formazan crystal formed at the bottom of the wells was dissolved with 200 µl DMSO for 20 minutes. The absorbance at 570 nm was read on a spectrophotometric plate reader (Hidex). The proportion of surviving cells was calculated as:



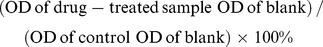
.

Dose-response curves were constructed to obtain the IC_50_ values. Experimental data were derived from 3 independent experiments. The selectivity index was obtained by mean IC_50_ RWPE-1/mean IC_50_ of LNCaP or PC-3.

### Cellomics Multiparameter Assay

Cytotoxicity 3 kit (Thermo Scientific) was used as described previously [25]. Briefly, 24 hours after subditine (**1**) treatment, MMP dye and the cell permeability dye were added to live cells and incubated for 30 minutes at 37°C. Cells were fixed, permeabilized, blocked with 1x blocking buffer before probing with primary cytochrome c and secondary DyLight 649 conjugated goat anti-mouse IgG antibodies for 1 hour each. Hoechst 33342 was added into the staining solution. Plates were then analyzed using the ArrayScan high content screening (HCS) system (Cellomics, PA, USA). Data were captured, extracted and analyzed with ArrayScan II Data Acquisition and Data Viewer version 3.0.

### ROS Assay

The production of intracellular ROS was detected as described previously [26]. The DHE dye reagent is converted to fluorescent ethidium and intercalates into DNA in response intracellular ROS. Briefly, 10 mM DHE stock solution (in methanol) was diluted 500-fold in HBSS without serum or other additives to yield a 20 µM working solution. After exposure to subditine (**1**), the cells in the 96-well black plate was washed twice with HBSS and then incubated in 100 µL working solution of DHE at 37°C for 30 minutes. Fluorescence of DCF in each cell was captured, extracted and analyzed with ArrayScan II Data Acquisition and Data Viewer version 3.0 (Cellomics).

### Gene Expression Profiling

LNCaP and PC-3 cells were treated with subditine (**1**) (12.5 µM) for 18 h. RNA was extracted from PC-3 or LNCaP cells using RNeasy plus mini kit (Qiagen). 1 µg of RNA was reverse transcribed into cDNA using the RT2 first strand kit (SA Biosciences, Qiagen).cDNA was mixed with RT2 Real Time™ SYBR Green/fluorescein PCR master-mix and loaded into each 96 wells of the Human Oxidative Stress and Antioxidant Defense qPCR array according to the manufacturer’s protocol (SA Biosciences, Qiagen). Briefly, a total volume of 25 µl of PCR mixture, which included 12.5 µl of mastermix, 11.5 µl of double distilled water, and 1 µl of cDNA was loaded into each of the 96wells. qPCR were done using StepOne PLUS real-time PCR machine (Applied Biosystems). PCR amplification was conducted at 95°C for 10 min, followed by 40 cycles of 95°C for 15 sec and 60°C for 1 min. The mRNA expression for each gene was normalized using the average expression of five housekeeping genes:

β-actin (ACTB), β-2-microglobulin (β2M), hypoxanthine phosphoribosyltransferase 1 (HPRT1), ribosomal protein L13a (RPL13A) and glyceraldehyde-3-phosphate dehydrogenase (GAPDH). The ΔΔCt method was used for data analysis. Fold changes were calculated for each gene as the difference in gene expression between subditine (**1**) or non-treated control using the RT Profiler qPCR-array data analysis software.

### Real-time qPCR Analysis

Total RNA was extracted from PC-3 or LNCaP cells using the RNeasy plus mini kit (Qiagen). RNA (1 µg) was reverse transcribed into cDNA using iScript cDNA synthesis kit (Biorad). QPCR was performed on the StepOne PLUS real-time PCR machine (Applied Biosystems) using SsoFast EvaGreen Supermix (Bio-Rad) according to the manufacturer’s protocols. Primers were commercially synthesized by Integrated DNA Technologies (IDT). Target mRNA values were normalized using β-actin mRNA and data were expressed relative to normalized values of corresponding controls. Samples were analyzed in three independent experiments in triplicates. Primers used were listed below,

GR Forward primer, AACATCCCAACTGTGGTCTTCAGC

GR Reverse primer, TTGGTAACTGCGTGATACATCGGG

β-actin Forward primer, GATGACCCAGATCATGTTTGAGACC

β-actin Reverse primer, AGTCCATCACGATGCCAGTGGT

### Bioluminescent Assays of Caspase-3/7,-8 and -9

A time-dependent study of caspase-3/7, -8, and -9 activities was performed in triplicates using assay kits Caspase-Glo 3/7, 8, and 9 (Promega Corp.,Madison,WI, USA) on white 96-well microplate as described previously [27]. A total of 10,000 cells per well was seeded and treated with 12.25 µM of subditine (**1**) for 6, 12, 18, 24, and 30 hours. Then, 100 µL of the caspase-Glo reagent was added, incubated at room temperature for 30 minutes and measured using Tecan Infinite 200 Pro (Tecan, Mannedorf, Swizerland) microplate reader.

### Western Blotting

To determine protein expression, 1*×*10^6^ cells/mL were seeded and treated with subditine (**1**) or paclitaxel for 24 h. Whole cell extracts were prepared as previously described [28]. Briefly, cells were collected, lysed and resolved on 10% SDS-polyacrylamide gels. After electrophoresis, the proteins were transferred to PVDF membranes (Millipore), blocked with 5% nonfat dry milk in PBS-T (0.05% Tween 20) for 1 hour at room temperature. Membranes were probed with primary rabbit anti-Bcl-2, Bcl-xL or p53 antibodies followed by horseradish peroxidase (HRP)-conjugated secondary anti-rabbit antibody (Cell Signaling Technology Inc., CA, USA). Membranes were stripped and reprobed with mouse anti-*β*-actin antibody as loading control (Santa Cruz Biotechnology Inc.). Protein-antibody complexes were detected with Amersham ECL prime Western blotting detection reagent (GE Healthcare, USA).

### Statistical Analysis

All values were expressed as mean ± S.D. Statistical analyses were evaluated by Student’s t-test. Probability values *p<0.05 was considered statistically significant.

## Results and Discussion

The dichloromethane extract of the bark of *Nauclea subdita* was subjected to column chromatography over silica gel 60 with gradient elution system of dichloromethane (CH_2_Cl_2_) and methanol (MeOH), giving 15 fractions. Further purification of the fractions using preparative thin layer chromatography yielded subditine (**1**) and four known alkaloids; angustoline (**2**), angustidine (**3**), angustine (**4**), nauclefine (**5**). Structural identification of **1** was done by 1D-, 2D-NMR, UV, IR and LCMS while the structure of known compounds (**2**–**5**) was identified through the comparison of NMR data with literature values.

### Characterization of Subditine (1)

Subditine (**1**) was isolated as a yellowish amorphous solid. The LCMS-IT-TOF spectrum revealed a pseudomolecular ion peak [M+H]^+^ at *m/z* 330.1018, corresponding to the molecular formula of C_20_H_15_N_3_O_2_ (calc. 330.1237). The IR spectrum of **1** showed an absorption band at 1645 cm^−1^, indicative of a conjugated lactam carbonyl functionality [18].

**Figure 2:**
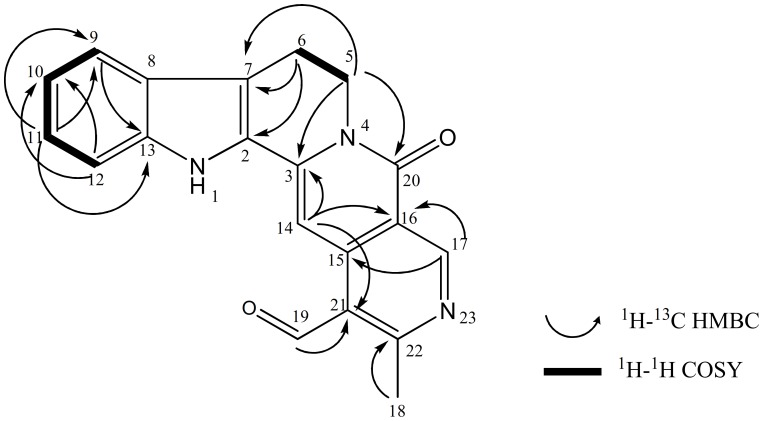
^1^H-^1^H COSY and HMBC correlations of subditine (1).

In the ^1^H-NMR spectrum, the presence of two doublets at δ_H_ 7.62 (1H, *d*, *J* = 7.8 Hz, H-9) and δ_H_ 7.47 (1H, *d*, *J* = 8.2 Hz, H-12), two doublet of doublets at δ_H_ 7.34 (1H, *dd*, *J* = 8.2, 7.1 Hz, H-11) and δ_H_ 7.19 (1H, *dd*, *J* = 7.8, 7.1 Hz, H-10), two methylenes at δ_H_ 4.51(1H, *m*, H-5) and δ_H_ 3.16 (1H, *m*, H-6), suggesting a naucleamide derivative with substitution pattern in ring A and C [29]. Furthermore, this tetrahydro-*β*-carboline skeleton (ring A, B, and C) was indicated with HMBC correlations of H-5 to C-3 (δ_C_ 139.4) and C-7 (δ_C_ 117.1), H-6 to C-2 (δ_C_ 127.3) and C-7, H-9 and H-11 to C-13 (δ_C_ 138.7) (Figure 2). A broad singlet at δ_H_ 8.94 implied the presence of an NH unit. The ^13^C-NMR and DEPT spectra of **1** indicated a total of twenty carbon signals; one methyl, two methylene, six methane, nine quaternary carbon and two carbonyl (Table 1). The carbonyl of the lactam ring resonated at δ_C_ 161.7. In addition, the HMBC spectrum showed correlation between H-14 (δ_H_ 7.97) and C-3, H-14 and C-16 (δ_C_ 119.3), H-5 (δ_H_ 4.51) and C-20 (δ_C_ 161.7), thus supporting the presence of a δ lactam ring. Furthermore, HMBC correlations of H-14 to C-16 and C-21 (δ_C_ 127.6), H-17 (δ_H_ 9.57) to C-15 (δ_C_ 141.1) and C-16, H-18 (δ_H_ 2.98) to C-22 (δ_C_ 165.9), H-19 (δ_H_ 10.72) to C-21 (δ_C_ 127.6) indicated that ring D is connected to a nicotinaldehyde ring (ring E) with a methyl group forming a 2-methylnicotinaldehyde unit. Subditine (**1**), has a nauclefine type of skeleton [30], and it is very similar to the known compound, angustidine (**3**) except that the former has an additional carbonyl group at C-21. ^1^H and ^13^C values for both compounds are listed in Table 1. Complete ^1^H and ^13^C-NMR assignments were established by thorough analysis of COSY, HMBC, HSQC and NOESY data.

### Biological Assay

#### Subditine (1) potently inhibited cell-growth of LNCaP and PC-3 prostate cancer cells

The anti-cancer effect of dichloromethane crude, subditine (**1**), angustoline (**2**), angustidine (**3**), angustine (**4**), nauclefine (**5**) and were evaluated on human prostate cancer cells LNCaP and PC-3 by MTT assays. IC_50_ values (dose required to inhibit the proliferative response by 50%) for each compound was shown in Table 2. Subditine (**1**) showed great inhibitory effect towards LNCaP cells at IC_50_ 12.24±0.19 µM while IC_50_ for angustoline (**2**), angustidine (**3**), angustine (**4**), nauclefine (**5**) were 58.09±0.05 µM, 140.27±0.10 µM, 149.16±0.09 µM and 86.35±0.09 µM respectively. Similar findings were obtained on PC-3 cells, where subditine (**1**) exhibited the highest activity (IC_50_ = 13.97±0.32 µM) compared to the other compounds. These findings support that subditine (**1**) is the most potent cytotoxic compound among the five tested.

**Table 2:** Subditine (**1**) angustoline (**2**), angustidine (**3**), angustine (**4**), nauclefine (**5**) and standard drug paclitaxel screening on LNCaP and PC-3 human prostate cancer and RWPE human normal prostate epithelial cell-lines using MTT assays.

Compounds	IC_50_ values at 24 hours		
	LNCaP	PC-3	RWPE-1
Subditine	12.24±0.19 µM	13.97±0.32 µM	30.48±0.08 µM
Angustoline	58.09±0.05 µM	67.31±0.87 µM	65.94±0.04 µM
Angustidine	140.27±0.10 µM	84.91±1.48 µM	36.07±0.05 µM
Angustine	149.16±0.09 µM	121.59±3.73 µM	98.39±0.10 µM
Nauclefine	86.35±0.09 µM	92.07±1.28 µM	72.85±0.06 µM
Paclitaxel (standard)	1.27±0.04 µM	1.33±0.02 µM	1.58±0.06 µM

24 hours post treatment, MTT salt was dissolved with DMSO and the absorbance was measured with Hidex microplate reader at 570 nm.

Subsequently, we tested the cytotoxicity effect of subditine (**1**) on RWPE-1 (human normal prostate epithelial cells). MTT assay showed a higher IC_50_ value at 30.48±0.08 µM, indicating that subditine (**1**) is 2.5 and 2.2 folds more potent against LNCaP and PC-3 (selectivity index (SI): [LNCaP/PC-3] = 2.49/2.18) prostate cancer cells than the normal prostate cells; RWPE-1. In contrast, standard drug paclitaxel showed less selectivity (SI: [LNCaP/PC-3] = 1.24/1.19) by exhibiting IC_50_ values of 1.27±0.04 µM, 1.33±0.02 µM and 1.58±0.06 µM against LNCaP, PC-3 and RWPE-1 respectively.

#### Subditine (1) induced cytoskeletal rearrangement and nuclear fragmentation

**Figure 3:**
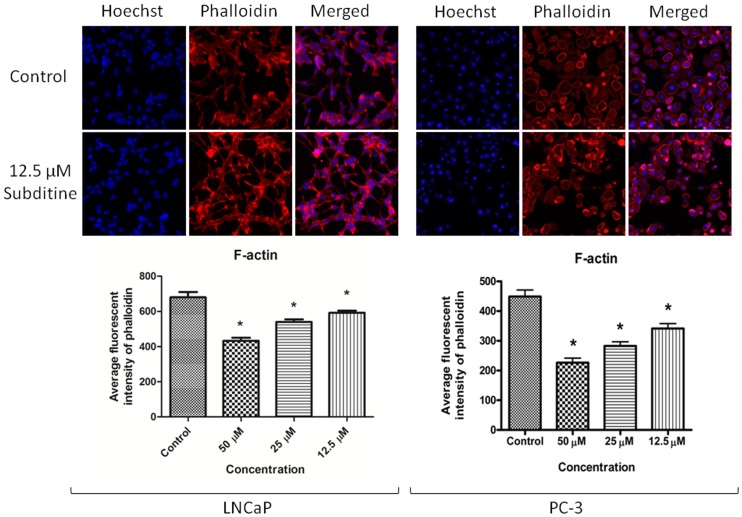
Subditine (1) induced cytoskeletal rearrangement at the peripheral. LNCaP and PC-3 cells were treated with subditine (**1**) at various concentrations for 24 hours. Cells were fixed and stained with Hoechst (blue) and phalloidin (red) dye which stained nucleus and polymerized actin (F-actin), respectively. Bar chart showing average fluorescent intensity of phalloidin (mean ± S.D.; *p<0.05). Dose-dependent increased of phalloidin intensity in LNCaP cells were observed after subditine treatment.

**Figure 4:**
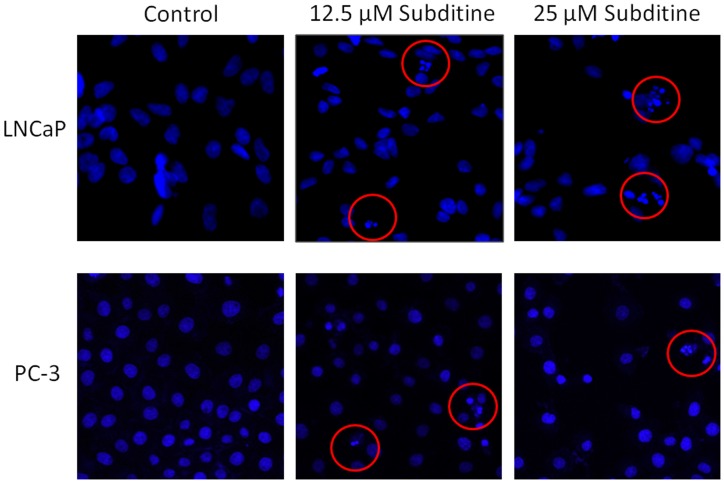
Subditine (1) treatment leads to nuclear DNA fragmentation. LNCaP and PC-3 cells were treated with subditine (**1**) (12.5 µM and 25 µM) for 24 h. Cells were then fixed and stained with Hoescht 33342 (blue). Red circles indicate DNA shrinkage or fragmentation. Images were captured using Cellomic arrayscan system.

Since subditine (**1**) significantly inhibited LNCaP and PC-3 cell growth, this compound was selected for further mechanistic studies. Cytoskeletal and nuclear morphological changes of LNCaP and PC-3 cells were examined by phalloidin (detects F-actin) and Hoechst 33342 staining. Results showed that some subditine (**1**) treated-cells displayed cell shrinkage with punctuate staining of F-actin at the peripheral membrane (Figure 3). At concentration 12.5 µM and 25 µM, nuclear condensation and fragmentation were detected at 24 hours after subditine (**1**) treatment (Figure 4). The nuclear intensity, corresponding to apoptotic chromatin changes were significantly increased following subditine (**1**) treatment in LNCaP and PC-3 cells (Figure 4, *P<*0.05). These results suggest that subditine (**1**) treatment induced apoptosis in LNCaP and PC-3 prostate cancer cells.

#### Subditine (1) promoted Reactive Oxygen Species (ROS) production

**Figure 5:**
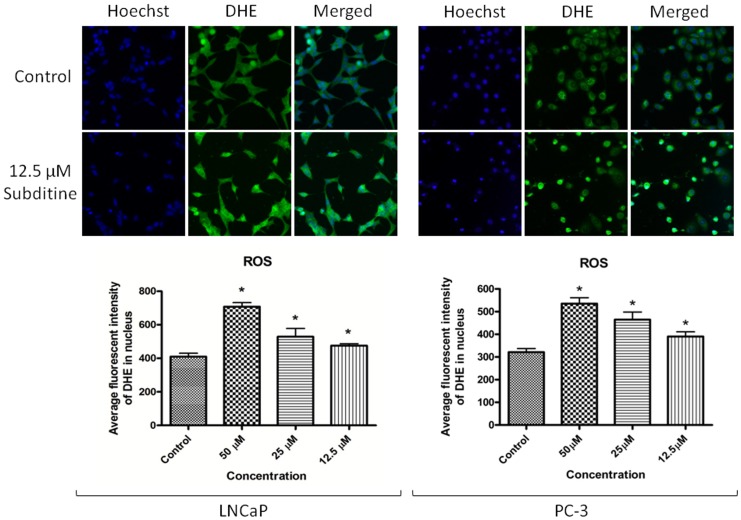
Subditine (1) enhanced ROS production in LNCaP and PC-3. LNCaP and PC-3 cells were treated with subditine (**1**) (12.5 µM, 25 µM, 50 µM) for 24 h. Cells were then fixed and stained with DHE dye. ROS levels were indirectly determined by measuring DHE dye incorporation in the nuclear using Cellomic HCS arrayscan. Increased DHE dye intensity in the nucleus was detected upon treatment of subditine. Hoechst (blue) and DHE dye (green). Bar chart showing the average fluorescent intensity of DHE stain (mean ± S.D.; *p<0.05).

ROS are natural by-products of the normal metabolism of oxygen. However, ROS level can increase dramatically upon environmental or chemical stress (e.g., presence of cytotoxic agent). To examine whether exposure of subditine (**1**) promotes ROS production, we stained live cells with DHE dye, 24 hours after subditine (**1**) treatment. DHE is rapidly oxidized to DCF by ROS and the fluorescent intensities were quantified with Cellomics High Content Screening. As shown in Figure 5, the levels of DCF fluorescence in LNCaP and PC-3 cells treated with subditine (**1**) were significantly increased in a dose-dependent manner.

Association between prostate cancer risk and oxidative stress has been well-recognized. There are considerable evidences suggesting oxidative stress contributes to the etiology and pathogenesis of the prostate cancer. Given that the mitochondria are a major source of ROS, altered mitochondrial bioenergetics might underlie the development of prostate cancer. Furthermore, high levels of ROS have been detected in several human cell-lines as well as in different human tissue. Some supporting evidences suggest that increased ROS generation could be a result of oncogenic transformation. Inherent oxidative stress may affect several functions in cancer cells or tumor tissue, such as cell proliferation, promotion of mutations and genetic instability, alterations in cellular sensitivity to anti-cancer agents, invasion and metastasis. Targeting ROS production rather than ROS neutralization might offer a novel mechanism in combating prostate cancer and perhaps other malignancies.

#### Subditine (1) induced gluthatione reductase gene expression

As we showed that subditine (**1**) has shown that it could induce ROS in cells, we decided to use human oxidative stress and antioxidant defense real time profiler qPCR-array to quantify gene expression changes in PC-3 or LNCaP cells treated with subditine (**1**). This qPCR-array contains 84 genes involved in cellular stress response and redox control and includes all six members of antioxidant peroxiredoxin (PRDX) family.

**Figure 6:**
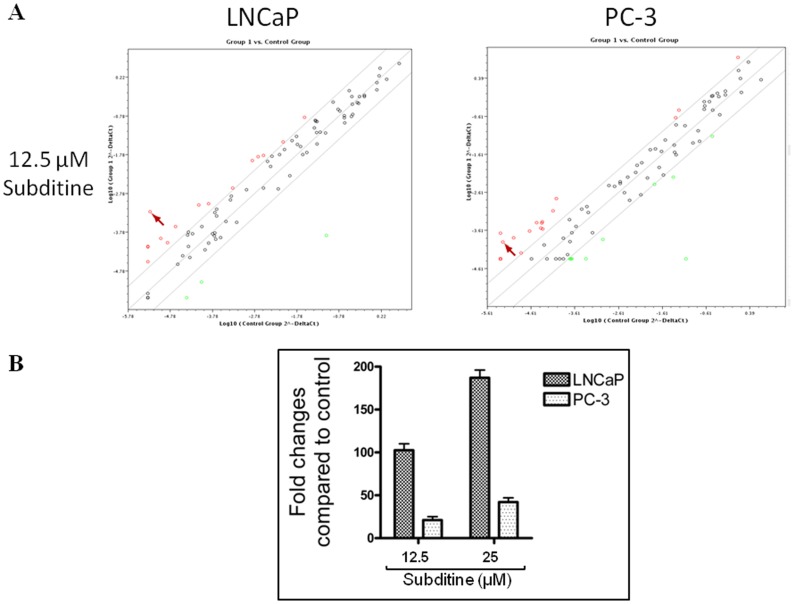
Subditine (1) induced glutathione reductase (GR) gene expression. LNCaP and PC-3 cells were treated with subditine (**1**) (12.5 µM) for 18 h. (A) Human oxidative stress and antioxidant defence qPCR-array was used to identify genes significantly up- or down-regulated in subditine (**1**)-treated LNCaP or PC-3 cells. Gene profiling analyses were performed three times in independent experiments. (Arrow indicates location of GR in the scatter plots) (B) Transcriptional changes of GR were evaluated using quantitative real-time-PCR. Levels of GR mRNA were normalized using β-actin housekeeping gene and expressed as fold change in comparison to untreated control.

The oxidative stress and antioxidant-related genes are differentially expressed in PC-3 or LNCaP cells in response to subditine (**1**). Interestingly, we noticed that glutathione reductase (GR) was significantly up-regulated (*P*<0.05) in both prostate cancer cell-lines relative to control cells (Fig. 6A). The fold change is more drastic in LNCaP (>100-fold) compared to PC-3 (>20-fold) cells. Subsequent independent qPCR analysis also showed that this gene is up-regulated in subditine (**1**)-treated cells, consistent with our qPCR array results (Fig. 6B).

GR is an important enzyme involved in the scavenging of active oxygen species. Our results suggest that enhanced ROS production by subditine (**1**) could stimulate GR *de novo* synthesis. GR is well-known for its anti-oxidant function and usually used as an indicator for oxidative stress. Upregulation of GR could be one of the cellular anti-oxidant defense mechanism in response to increasing ROS. However, superfluous generation of reactive oxygen species could overwhelm the antioxidant system, which triggers a cascade of events that leads to lipid-protein damage, uncoupling the oxidative phosphorylation and eventually results in apoptosis.

#### Subditine (1) increased membrane permeability, reduced Mitochondrial Membrane Potential (MMP) and increased cytochrome c release

**Figure 7:**
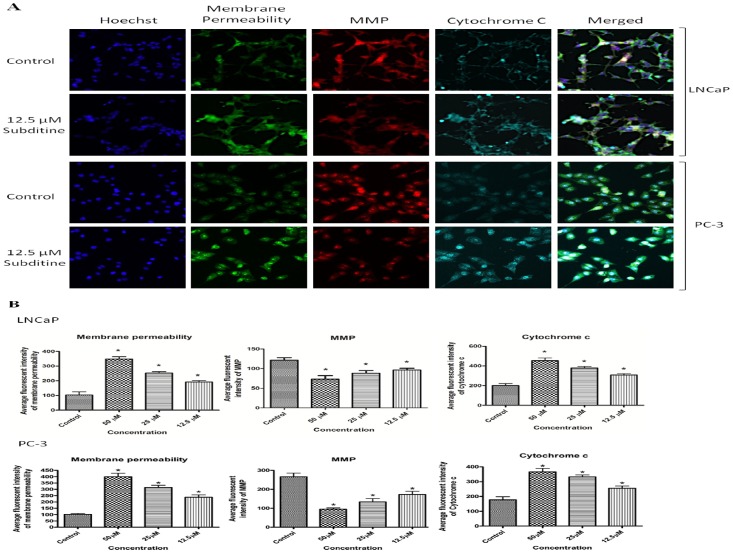
Dose-dependent effect of subditine (1) on cell membrane permeability, MMP and cytochrome c release. LNCaP and PC-3 cells were treated with subditine (**1**) for 24 h. Cells were then fixed and stained with membrane permeability dye, MMP, cytochrome c and Hoechst as described in Materials and Methods. (A) Stained cells were visualized using HSC arrayscan system to check nuclear morphology, membrane permeability, MMP integrity, cytochrome c release; Blue (nuclear), Green (Membrane permeability), Red (MMP), Cyan (cytochrome c release). (B) Bar chart showing the average fluorescent intensities of membrane permeability, MMP and cytochrome c (mean ± S.D.; *p<0.05).

To get a better insight into the mechanism of subditine (1)-induced cytotoxicity, the changes in membrane permeability, mitochondrial membrane potential (MMP) and cytochrome c localization after subditine (1) treatments were measured. As expected, subditine (1)-treated LNCaP and PC-3 cells demonstrated higher membrane permeability as compared to control as most treated cells undergo apoptosis due to cytotoxic activity of the compound (Figure 7A and 7B). In addition, results showed that subditine (1) treatment caused loss of MMP, suggesting a plausible mechanism for cell death. As shown in Figure 6A, MMP dye stained strongly in the cytoplasm of control cells compared to subditine (1)-treated cells. LNCaP and PC-3 cells treated with subditine (1) for 24 hours showed dose-dependent reduction of MMP fluorescence intensity (Figure 7B), which reflected the collapse of MMP. On the other hand, subditine (1) treated-LNCaP and PC-3 showed increased fluorescent-staining in the cytosol compared to control, indicating cytochrome c release (Figure 7A and 7B). These results suggest that subditine (1) triggered the loss of MMP and subsequent translocation of cytochrome c from mitochondria into the cytosol in LNCaP and PC-3 cells.

#### Subditine (1) activated caspase 9 and 3/7

**Figure 8:**
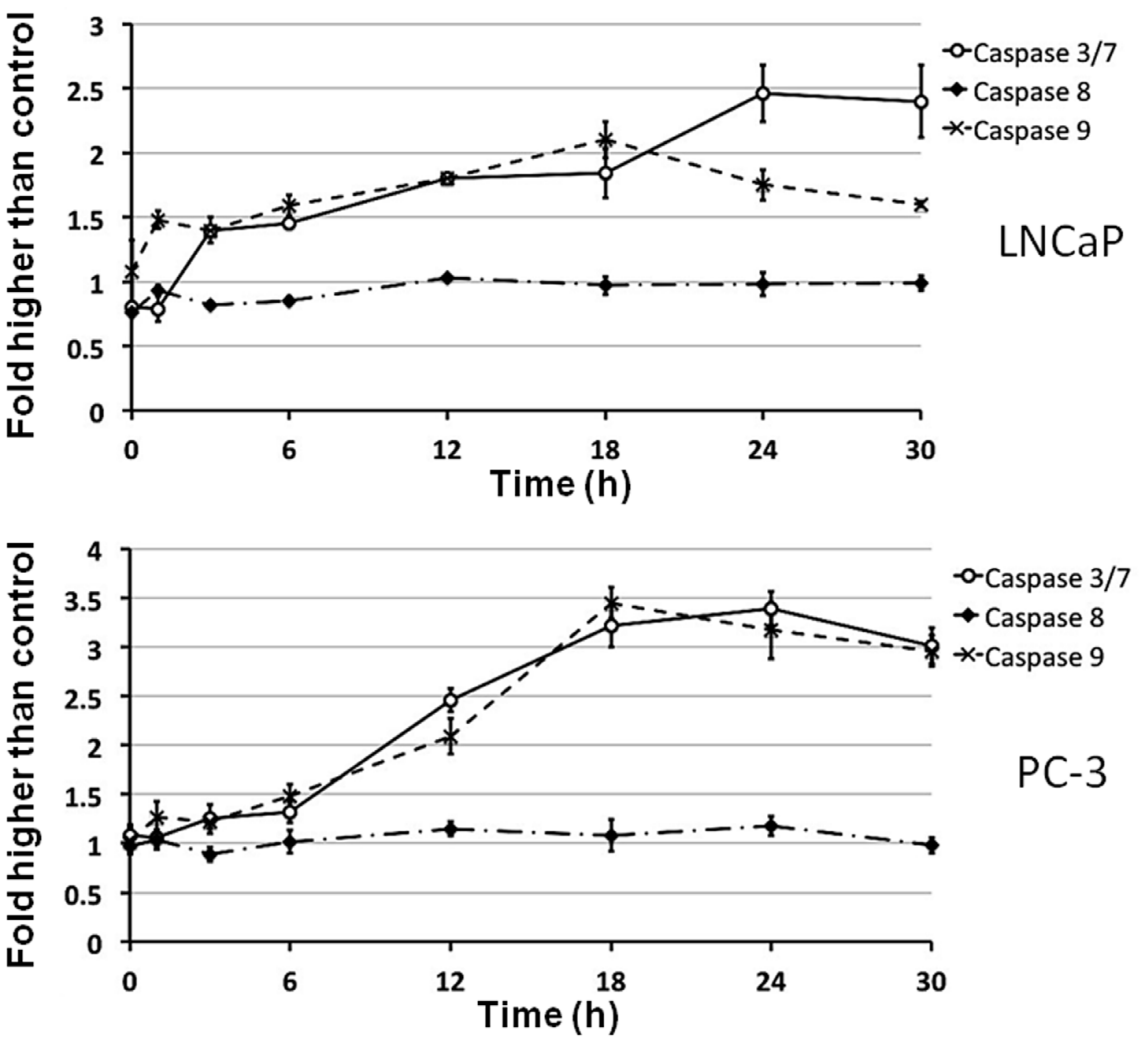
Subditine (1) induced caspase 9, 3/7 activation in LNCaP and PC-3 cells. LNCaP and PC-3 cells were treated with subditine (12.5 µM) and caspase 8, 9, 3/7 activities were determined using bio-illuminescent assays at the indicated time point. Subditine (**1**) induced caspase 9, 3/7 activation in both LNCaP and PC-3 cells. No significant fold-change was detected in caspase 8 activity throughout 30 hours.

The release of cytochrome c from mitochondria activates downstream caspase molecules and lead to apoptotic cell death. To examine this, the bioluminescent intensities of caspase-3/7, -8, -9 activities of subditine (**1**)-treated LNCaP and PC-3 cells at 6, 12, 18, 24, or 30 hours time-points were measured. As shown in Figure 8, significant increase in caspase-3/7, -9 activities were detected in both LNCaP and PC-3 cells after 12 and 24 hours of subditine (**1**) exposure. The highest activity for caspase-9 in both cell lines was observed after 24 hours of treatment with subditine (**1**). On the other hand, caspase-3/7 activity reached to a peak after 18 hours of treatment and gradually decreased at later time points (24 and 30 hours). Neither LNCaP nor PC-3 cells exhibited any induction of caspase-8 activity during 30 hours of subditine (**1**) treatment. Thus, these data suggest that subditine (**1**)-induced apoptosis in LNCaP and PC-3 is mediated via the intrinsic (mitochondrial- caspase-9) pathway, but not extrinsic (death receptor caspase-8) pathway.

Caspases are recognized as universal effectors in apoptotic cell death. Apoptotic signals triggers the initiator caspases such as caspase 2, 8, 9 and 10, which in turn induce activation of other caspases [31], [32]. In this study, cytochrome c was translocated from mitochondria to the cytosol upon treatment with subditine (**1**). The release of cytochrome c forms apoptosome via interaction with Apaf-1, pro-caspase-9 and Bcl-xL. The apoptosome activates caspase-9, which in turn activates downstream caspases, including caspases -3, -6 and -7. The execution of caspase cascade 3/7 and -9 through mitochondria signaling pathway has been demonstrated to be an efficient way of killing prostate cancer cells [31], [33].

#### Effect of subditine (1) on Bcl-2, Bcl-xL and p53 expression

**Figure 9:**
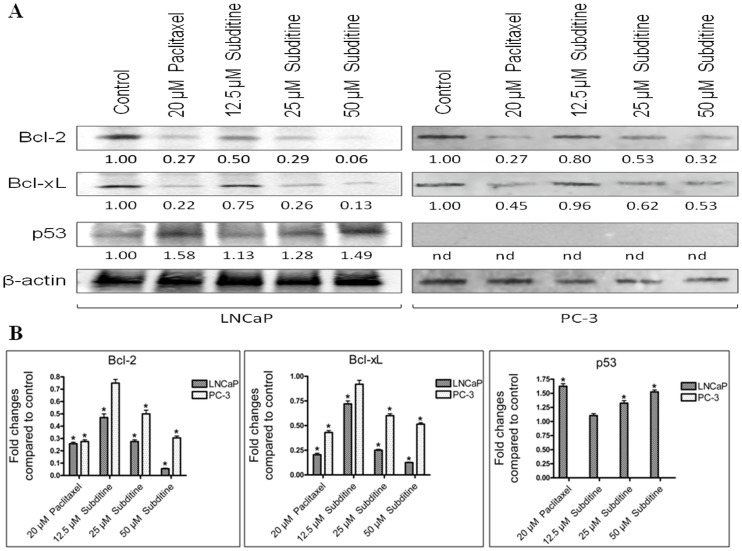
Western blotting analyses of apoptosis-associated molecules after subditine (1) treatment. LNCaP and PC-3 cells were treated with paclitaxel (positive control) or various concentrations of subditine (**1**) for 24 hours. Cells were lysed, subjected to SDS-PAGE and Western blotting. (A) Membranes were probed with Bcl-2, Bcl-xL and p53 antibodies. Protein loading was assessed with antibody to β-actin. Normalization for loading differences was done by dividing the densitometry values for individual bands with β-actin in the same lane (n.d.-not determined). (B) Bar charts showing densitometry quantification of Bcl-2, Bcl-xL and p53 expression in subditine (**1**)-treated cells relative to control (mean ± S.D.; *p<0.05).

It is well established that anti-apoptotic proteins (Bcl-2, Bcl-xL, p53) play an important role in maintaining MMP and preventing apoptosis in cancer cells. To determine the underlying mechanism of subditine (**1**)-induced apoptosis, the expressions of apoptosis-related proteins were investigated. In subditine (**1**)-treated PC-3 samples, Western blot results showed a dose-dependent reduction of Bcl-2 and Bcl-xL expression levels. In PC-3 cells, Bcl-2 and Bcl-xL protein showed no significant change at 12.5 µM compare to control, but they were drastically down-regulated at 25 and 50 µM dosages (Figure 9A and B). In addition, subditine (**1**) could up-regulate expression of p53 in LNCaP, but not PC-3 cells (Figure 9A and B). This result is consistent with other study as PC-3 is devoid of p53 expression due to a frame-shift mutation [34]. Together, these data suggested that subditine (**1**) induced apoptosis via the mitochondrial-pathway by modulating the expression of anti-apoptotic molecules.

Bcl-2 is an anti-apoptotic mediator expressed in many cancer types, for instance prostate, breast, ovarian, lung, colon and renal cancers [35], [36]. It serves as checkpoint to execution of caspase cascade and mitochondrial dysfunction [37]. Bcl-xL, one of the members of Bcl-2 family, blocks cell death via regulation of mitochondrial homeostasis [36], [38]. Besides, Bcl-xL downregulation could be responsible for caspase-9 activation, due to the principal role of Bcl-xL/Apaf1 interaction in the inhibition of Apaf1-dependent caspase-9 activation [39]. In contrast, over-expression of Bcl-2 and Bcl-xL has been associated with the progression of prostate cancer and protection of prostate cancer cells against various therapeutic interventions such as hormone ablation, radiotherapy and chemotherapy [40]–[42]. For instance, Lebedeva et al. have shown that up-regulation of Bcl-2 could inhibit p53-induced apoptosis in LNCaP cells [41]. Besides, Lebedeva et al. found that tumors that initially responded well to chemotherapy, could develop into resistant clones due to increase protein levels of Bcl-2 or Bcl-xL [43].

Tumour suppressor protein, p53 is a mediator of apoptosis in many cells and triggers apoptosis in response to DNA damage [44]. The activation of cell death pathway is important to remove irreparable damaged cells [45]. In contrast, down-regulated or dysfunctional p53 may induce tumour progression and resistance to chemotherapy. For instance, a report by Rokhlin showed that p53 inactivation in prostate cancer cell-line (LN-56) is associated with increase resistance to TNF-α treatment [46]. Therefore, reactivation or enhanced p53 expression in cancer cells plays new role in therapeutic measure [47]. Here, we showed that subditine (**1**) induces apoptosis in LNCaP cells through p53 up-regulation, coupled with Bcl-2 and Bcl-xL down-regulation. However, subditine (**1**) mediated cell death in PC-3 cells was independent of p53 expression. Nevertheless, we showed that subditine (**1**) did not restore or enhance p53 expression. Thus, we hypothesized that targeting Bcl-2 family proteins such as Bcl-2 or Bcl-xL could be of therapeutic values in p53-negative prostate cancer cells.

## Conclusion

**Figure 10:**
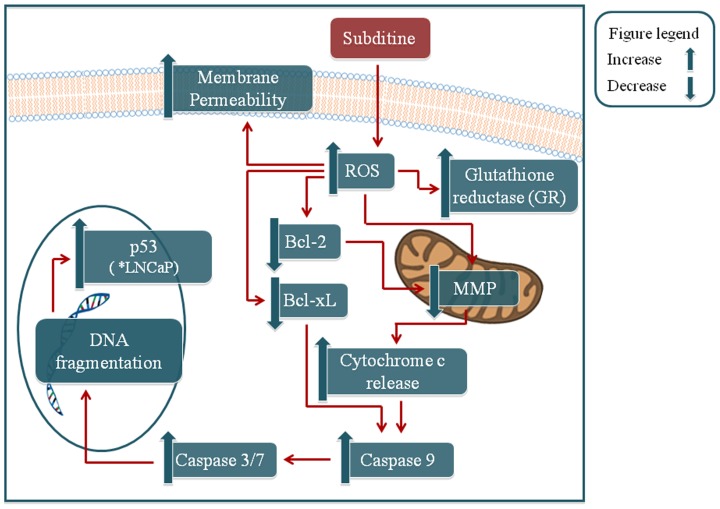
Schematic diagram of possible mechanism of subditine (1)-induced apoptosis in LNCaP and PC-3 cells. Subditine (**1**) treatment leads to increase ROS production, which induced GR expression. Excessive ROS disrupts the MMP, promotes cytochrome c release from mitochondria and activates downstream caspase 9, 3/7. We also showed that subditine (**1**) modulates the expression of apoptotic regulatory proteins (Bcl-2, Bcl-xL, p53 (*in LNCaP)) involved in the complex intrinsic apoptosis signal cascades. Apoptosis is evident through increased cell permeability and DNA fragmentation. Overall, subditine (**1**) mediated cell death through ROS-induced signal-transduction cascades and activation of a set of caspases.

In conclusion, our report suggest that subditine (**1**), a new monoterpenoid indole alkaloid from bark of *Nauclea subdita* significantly inhibited prostate cancer LNCaP and PC-3 cell-growth by inducing apoptosis as evidenced by cytoskeletal rearrangement and nuclear DNA fragmentation. In addition, subditine (**1**) also exhibited better selectivity index (2.49) compared to the standard drug paclitaxel (1.24). Mechanistic studies showed that subditine (**1**) treatment promotes ROS production, as reflected by increase GR expression. Excessive ROS reduces MMP, which in turn stimulates cytochrome c release from mitochondria (Figure 10). Cytosolic cytochrome c activates caspase 9 and 3/7, which triggers the apoptotic machinery (Figure 10). Further study revealed that subditine (**1**) induces Bcl-2 and Bcl-xL down-regulation in both prostate cancer cell-lines, indicating mitochondrial-mediated apoptosis pathway. Subditine (**1**) treatment also leads to higher p53 expression in LNCaP, but not in PC-3 cells. These findings provide new insights on the potential anti-cancer property of subditine (**1**) in human prostate cancer, which should be followed up in future studies using *in vivo* animal model.
